# Women’s health behaviour change after receiving breast cancer risk estimates with tailored screening and prevention recommendations

**DOI:** 10.1186/s12885-022-09174-3

**Published:** 2022-01-16

**Authors:** Linda Rainey, Daniëlle van der Waal, Louise S. Donnelly, Jake Southworth, David P. French, D. Gareth Evans, Mireille J. M. Broeders

**Affiliations:** 1 Radboud Institute for Health Sciences, Radboud University Medical Center, PO Box 9101, 6500 HB Nijmegen, The Netherlands; 2 Dutch Expert Centre for Screening, PO Box 6873, 6503 GJ Nijmegen, The Netherlands; 3 Centre for Mental Health and Safety, University of Manchester, Manchester, M13 9PL England; 4 Prevent Breast Cancer Research Unit, The Nightingale Centre, Manchester University NHS Foundation Trust, Southmoor Road, Manchester, M23 9LT UK; 5 Manchester Centre for Health Psychology, School of Health Sciences, University of Manchester, Coupland Street, Manchester, M13 9PL UK; 6 Genomic Medicine, Division of Evolution and Genomic Sciences, Manchester Academic Health Sciences Centre, Manchester University NHS Foundation Trust, Manchester, M13 9WL UK; 7 The Christie NHS Foundation Trust, Withington, Manchester, M20 4BX UK

**Keywords:** Breast cancer, Risk assessment, Screening, Prevention, Uptake

## Abstract

**Background:**

The Predicting Risk of Cancer at Screening (PROCAS) study provided women who were eligible for breast cancer screening in Greater Manchester (United Kingdom) with their 10-year risk of breast cancer, i.e., low (≤1.5%), average (1.5–4.99%), moderate (5.-7.99%) or high (≥8%). The aim of this study is to explore which factors were associated with women’s uptake of screening and prevention recommendations. Additionally, we evaluated women’s organisational preferences regarding tailored screening.

**Methods:**

A total of 325 women with a self-reported low (*n* = 60), average (*n* = 125), moderate (*n* = 80), or high (*n* = 60) risk completed a two-part web-based survey. The first part contained questions about personal characteristics. For the second part women were asked about uptake of early detection and preventive behaviours after breast cancer risk communication. Additional questions were posed to explore preferences regarding the organisation of risk-stratified screening and prevention. We performed exploratory univariable and multivariable regression analyses to assess which factors were associated with uptake of primary and secondary breast cancer preventive behaviours, stratified by breast cancer risk. Organisational preferences are presented using descriptive statistics.

**Results:**

Self-reported breast cancer risk predicted uptake of (a) supplemental screening and breast self-examination, (b) risk-reducing medication and (c) preventive lifestyle behaviours. Further predictors were (a) having a first degree relative with breast cancer, (b) higher age, and (c) higher body mass index (BMI). Women’s organisational preferences for tailored screening emphasised a desire for more intensive screening for women at increased risk by further shortening the screening interval and moving the starting age forward.

**Conclusions:**

Breast cancer risk communication predicts the uptake of key tailored primary and secondary preventive behaviours. Effective communication of breast cancer risk information is essential to optimise the population-wide impact of tailored screening.

**Supplementary Information:**

The online version contains supplementary material available at 10.1186/s12885-022-09174-3.

## Background

**Fig. 1:**
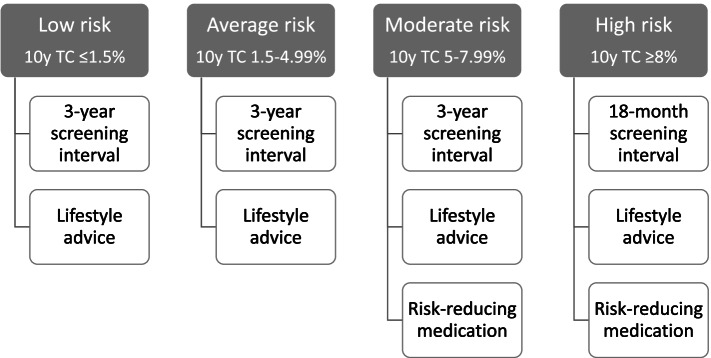
Risk-tailored screening and prevention pathways of the PROCAS study

In the United Kingdom (UK), the National Health Service Breast Screening Programme (NHSBSP) offers triennial mammography screening to women aged 50–70 years [1]. It effectively enables early detection of breast cancer, thereby potentially improving treatment options and reducing breast cancer mortality [2]. The balance of screening benefits against known harms (overdiagnosis, overtreatment, false-positive recall) may improve when screening is based on a woman’s individual breast cancer risk. The Predicting Risk of Cancer at Screening (PROCAS) study has shown that breast cancer risk assessment is feasible within the breast cancer screening setting of Greater Manchester, England [3]. To assess breast cancer risk, up to three sources of information were collected amongst 53,000 women between 2009 and 2013, i.e., (a) self-reported information on family history of breast cancer, parity, body mass index (BMI), height, age at menarche/menopause/first live birth, menopause hormone therapy use, (b) mammographic density, and (c) single-nucleotide polymorphisms (SNPs) derived from saliva. With this information, women could be classified as low, average, moderate, or high risk of developing breast cancer within the next 10 years using the Tyrer-Cuzick (TC) risk prediction model [4]. Screening and primary prevention recommendations were subsequently provided to women based on their estimated breast cancer risk (Fig. 1). High-risk women were offered more frequent screening. Additionally, moderate and high-risk women were informed about taking medication (tamoxifen or raloxifene) to potentially decrease their breast cancer risk [5]. All risk categories were recommended to maintain a healthy lifestyle characterised by limited alcohol intake, a Mediterranean diet, and physical activity levels in line with cancer prevention guidance [6].

For risk-based screening to have a real impact on breast cancer incidence, uptake of screening and prevention recommendations needs to be optimal. However, evidence suggests that communicating personalised breast cancer risk has no consistent effects on women’s intentions to change screening or prevention behaviours [7]. This finding is consistent with previous research showing that personalised risk estimates alone do not have much impact on health-related behaviour change [8, 9]. However, when risk information is given in conjunction with information about how to reduce the risk, it has larger effects on behaviour [10]. It additionally appears that risk perceptions more strongly impact motivations to change early detection behaviours, e.g., screening [11], than prevention behaviours, e.g., uptake of a weight loss programme [12]. This highlights the importance to evaluate the uptake of early detection and prevention behaviours separately.

The present research therefore aimed to explore which factors predict PROCAS participants’ uptake of screening and prevention recommendations after breast cancer risk communication. Additionally, we assessed their preferences regarding the organisation of risk-stratified breast cancer screening and prevention.

## Methods

### Design

Cross-sectional data were collected between February and April 2019 in the Greater Manchester Area (UK) using a web-based survey which was designed using qualitative focus group data [13]. Ethics approval was acquired from the London Central NHS Research Ethics Committee (16/LO/0925). Informed consent was obtained online prior to the start of the survey.

### Participants

Women were selected from the participant database of the PROCAS study. All PROCAS participants had previously received their personal 10-year TC breast cancer risk category, i.e., low (≤1.5%), average (1.5–4.99%), moderate (5.-7.99%) or high (≥8%) risk, between October 2014 and June 2016. Each risk category contained tailored screening and preventive options based on the information materials of the PROCAS study (Fig. 1). For the survey study, women were randomly sampled within each risk category if they met the following inclusion criteria: meet the UK screening eligibility criteria at the time of the survey study, i.e. aged 50–70 years and without a breast cancer diagnosis, and consented to being approached for follow-up studies.

In January and February 2019 we sent 2800 women in the Greater Manchester Area a participant information sheet, i.e., 700 per risk category. Women could contact the study team by email or telephone if they wanted to participate in the study, after which they received an e-mail with a weblink to the online survey.

### Procedure

The survey took 20–30 min to complete and contained two parts. In the first part, all participants answered questions about different aspects of their lives, e.g., demographics, family history, and general health. For the second part, participants were asked to recall their breast cancer risk as counselled by the PROCAS study team, i.e., low, average, moderate, or high risk. We had to rely on self-reported breast cancer risk, since we did not have information on counselled risk at time of survey completion. Dependent on their self-reported risk (Fig. 1), women were asked about early detection behaviours, i.e., (1) supplemental mammography outside of the national screening programme, i.e., women acquiring a referral through their GP for a clinical mammogram in between screening mammograms; (2) increased breast self-examination; and uptake of preventive behaviours, i.e., (3) risk-reducing medication and (4) lifestyle changes (diet, physical activity, and alcohol intake). Additional questions were posed to explore preferences regarding the organisation of risk-stratified screening and prevention, i.e., acceptability of risk feedback in a letter, need for risk consultation, preferred risk counsellor, potential use of informative website, preferred screening interval, and preferred way of taking risk-reducing medication. A copy of the survey is available upon request.

### Measures

#### Outcome variables

Women were asked about changes to their early detection and preventive behaviours since receiving their breast cancer risk estimate, i.e., 31–52 months after risk counselling.

Early detection behaviours: (1) intent to request supplemental mammography outside the national screening programme (yes vs. no or do not know; for average and moderate risk groups), (2) increased breast self-examination (yes vs. no).

Preventive behaviours: (3) started with preventative medication (yes vs. no; for moderate and high-risk groups), (4) changed diet (yes vs. no), (5) changed physical activity levels (yes vs. no), (6) changed alcohol intake (yes vs. no).

#### Determinants

##### Self-reported breast cancer risk

Participants were asked what they remember their counselled breast cancer risk was, i.e., low, average, moderate, or high. Participants provided their PROCAS study ID which enabled us to obtain their actual counselled breast cancer risk after survey completion.

##### Sociodemographic variables

Data on participants’ age (continuous), educational attainment (lower education, higher secondary education, higher vocational education including university and post-graduate degree), marital status (living together versus alone), and body mass index (BMI in kg/m^2^, continuous) were acquired.

##### Medical history

We included information on women’s medical history, i.e. family history of breast cancer (yes/no), personal history of benign breast disease (yes/no), previous biopsy (yes/no), diagnosed with one of the following medical conditions: cardiovascular disease, stroke, high blood pressure, asthma, chronic bronchitis, COPD, diabetes, ulcer, kidney disease, liver disease, anaemia, thyroid disease, depression, arthritis, and backache (< 2, ≥ 2 to indicate co-morbidity), and current medication use for one or more of these conditions, including menopausal hormone therapy (yes/no).

##### General health

General health on the day of participation was measured with the general EuroQol visual analogue scale (EQ VAS) [14]. It records self-rated health on a vertical scale ranging from 0 (worst imaginable health state) to 100 (best imaginable health state). It was included in analyses as a continuous measure.

##### Health anxiety

Health anxiety was measured with the validated 14-item Short Health Anxiety Inventory [15, 16]. Each item contains four statements. Participants are asked to choose the statement that best describes their feelings from the past 6 months on a scale from 0 (low health anxiety) to 3 (high health anxiety). A total sum score was calculated which was used as a continuous measure in analyses.

##### Health locus of control

Health locus of control refers to a person’s beliefs or expectations about which persons or other factors determine their health [17]. It was measured with the widely-used and validated 18-item Multidimensional Health Locus of Control Scales [17, 18]. There are three scales of each six items assessing an internal locus of control, a powerful others locus of control, and a chance locus of control. Answers are provided on a 6-point Likert scale ranging from strongly disagree (1) to strongly agree (6). For each scale, a total score can be calculated by summing the items. For analyses we used the highest scale score for each participant to indicate their main locus of control, resulting in a variable with four categories, i.e. 1 ‘mostly internal’, 2 ‘mostly powerful others, e.g. physicians’, 3 ‘mostly chance’, and 4 ‘no clear preference’.

##### Life events

Life events experienced in the past year were measured using an abbreviated version of the validated Holmes-Rahe Stress Inventory [19]. Based on our previously described focus group results [13], we included 10 life events that were considered relevant to the potential adoption of risk-based breast cancer screening and prevention. For analyses, we used a cut-off score of < 2 and ≥ 2 life events in the past year to enable meaningful group analysis.

##### Beliefs about medicines

General beliefs about medicines were measured with the validated 8-item Beliefs About Medicines Questionnaire [20]. It comprises of two 4-item scales: the first scale assesses the belief that medicines are harmful, addictive, poisonous and should not be taken continuously; the second scale assesses the belief that medicines are overused by physicians. Answers are scored on a 5-point Likert scale ranging from 1 (strongly disagree) to 5 (strongly agree). Higher scores indicate a stronger belief. A sum score was calculated for both subscales and used continuously in analyses.

### Statistical analyses

We used multiple imputation to impute missing data on age (5.8%), education level (8.6%), BMI (6.5%), history of benign breast disease (3.4%), and previous breast biopsy (1.5%). Missing data on these variables were due to women omitting to supply an answer to these questions in the survey. We additionally imputed missing data on counselled breast cancer risk as relayed by the PROCAS study team (5.8%). These missing values were due to women omitting to supply an identification number and/or date of birth, which left us unable to link their survey information to PROCAS study records. Pattern analysis was performed to ensure that data was missing at random. All six variables with missing data were added to the multiple imputation model in addition to marital status, first degree family history of breast cancer, current medication use, mammography intent, supplemental mammography intent, performance of breast self-examination, uptake of preventive medication, and changes to diet, physical activity, and alcohol intake, which were used as indicators. A total of 10 imputed datasets were created using univariate regression with no rounding.

Descriptive statistics were presented to establish women’s general characteristics and their screening and prevention behaviours/preferences. These were further stratified by self-reported and counselled breast cancer risk (i.e., low, average, moderate, and high).

We performed exploratory univariable and multivariable logistic regression analyses to calculate odds ratios. These odds ratios and their 95%-confidence intervals were used to assess which factors are associated with women deciding to (1) request additional mammography outside the screening programme, (2) increase breast self-examination, (3) change dietary habits, (4) increase exercise habits, (5) reduce alcohol intake, and (6) start a course of preventative medication. The chosen determinants were based on a systematic review of the literature and our previous focus group study [13, 21]. In the multivariable analyses, all associations were corrected for confounding by adding all potential confounders to the model simultaneously. Results from the multivariable analyses are presented below. For results from the univariate analyses we refer you to Supplement 5. For our main analysis we relied on women’s self-reported breast cancer risk, since subsequent survey questions on screening and prevention pathways were based on this risk. We performed two sensitivity analyses (Supplement 1) assessing the main outcomes for women who correctly reported their counselled risk and for women who reported another risk than counselled. For both sensitivity analyses, we selected the determinants and outcomes with sufficient sample sizes. Overall, results were relatively similar for both groups. However, first degree family history of breast cancer was only a predictor of intent to request supplemental mammography in women who correctly reported their counselled risk. Analyses were performed with IBM SPSS version 22 (Armonk, NY: IBM Corp).

## Results

### Participants’ breast cancer risk

A total of 325 women participated in the study (response rate 11.6%). Women were, on average, 61 years of age, had completed secondary education or higher, and were living with a partner (Table 1). They had a self-reported low (*n* = 60), average (*n* = 125), moderate (*n* = 80), or high (*n* = 60) risk of developing breast cancer (Table 1). The distribution of breast cancer risk factors did not correspond to women’s self-reported risk. We therefore also stratified women’s characteristics by counselled breast cancer risk (Supplement 2). This shows a distribution of breast cancer risk factors more in line with established epidemiology, with a higher prevalence of a first degree family history of breast cancer and benign breast disease among women at increased risk. Supplement 3 shows the level of correspondence between women’s self-reported risk and counselled risk. Based on counselled risk, only 23.2% of participants had an average breast cancer risk, whereas 61.1% were at increased risk, showing response bias. This is confirmed by comparing the characteristics (e.g., family history of breast cancer) of our study sample with those of all PROCAS participants (*n* = 53,596; Supplement 4). For 72.4% (*n* = 97) of women who incorrectly recalled their counselled risk, this inaccuracy led to survey questions on screening and prevention recommendations that they would not have received. Conspicuously, only six participants reported this discrepancy in the open-ended comment box with which the survey concluded.

**Table 1:** General characteristics of all participants, and for each self-reported breast cancer risk category

	All women ***N*** = 325		Low risk ***N*** = 60		Average risk ***N*** = 125		Moderate risk ***N*** = 80		High risk ***N*** = 60	
Age (years), mean (SD)^a^	61.3	(4.9)	61.9	(4.7)	61.5	(5.1)	61.8	(4.9)	60.0	(4.6)
Education level, n (%)^b^										
Lower education	68	(20.9)	14	(23.3)	27	(21.6)	14	(17.5)	13	(21.7)
Higher secondary education	98	(30.2)	13	(21.7)	42	(33.6)	26	(32.5)	17	(28.3)
Higher vocational qualification	131	(40.3)	24	(40.0)	46	(36.8)	37	(46.3)	24	(40.0)
Marital status, n living with partner (%)	256	(78.8)	54	(90.0)	94	(75.2)	61	(76.3)	47	(78.3)
First degree family history breast cancer, n yes (%)	138	(42.5)	4	(6.7)	41	(32.8)	49	(61.3)	44	(73.3)
Body mass index (kg/m^2^), mean (SD)^c^	24.9	(3.5)	25.1	(3.3)	24.7	(3.5)	24.8	(3.4)	25.3	(3.8)
Medical condition, *n* ≥ 2 diagnosed (%)	164	(50.5)	27	(45.0)	68	(54.4)	47	(58.8)	22	(36.7)
Current medication use, n yes (%)	144	(44.3)	30	(50.0)	56	(44.8)	33	(41.3)	25	(41.7)
Current MHT^d^ use, n yes (%)	26	(8.0)	8	(13.3)	14	(11.2)	4	(5.0)	20	(33.3)
Benign breast disease, n yes (%)^e^	121	(37.2)	21	(35.0)	41	(32.8)	35	(43.8)	24	(40.0)
Previous breast biopsy, n yes (%)^f^	81	(24.9)	10	(16.7)	30	(24.0)	21	(26.3)	20	(33.3)
General health score, mean (SD)	82.7	(14.6)	84.1	(17.5)	81.3	(15.0)	84.4	(11.9)	81.7	(14.2)
Life events, n ≥ 2 (%)	89	(27.4)	15	(25.0)	34	(27.2)	24	(30.0)	16	(26.7)
Health locus of control^g^, n (%)										
Internal	109	(33.5)	21	(35.0)	44	(35.2)	23	(28.7)	21	(35.0)
Physician	11	(3.4)	2	(3.3)	5	(4.0)	2	(2.5)	2	(3.3)
Chance	167	(51.4)	31	(51.7)	61	(48.8)	47	(58.8)	28	(46.7)
No clear preference	38	(11.7)	6	(10.0)	15	(12.0)	8	(10.0)	9	(15.0)
Belief in medicines, mean (SD)										
Harm	7.9	(2.3)	8.5	(2.6)	7.8	(2.2)	8.0	(2.2)	7.3	(2.2)
Overuse	11.3	(3.0)	11.6	(3.0)	11.2	(2.8)	11.6	(3.2)	10.9	(3.0)
Health anxiety, mean (SD)	10.8	(4.9)	8.8	(4.4)	10.8	(4.9)	11.2	(4.3)	12.2	(5.4)

^a^ *n* = 19 missing values (5.8%); ^b^ *n* = 28 missing values (8.6%); ^c^ *n* = 21 missing values (6.5%); ^d^ Menopause hormone therapy; ^e^ *n* = 11 missing values (3.4%); ^f^ *n* = 5 missing values (1.5%); ^g^ *HLoC* health locus of control

### Early detection behaviours after risk communication

Table 2 describes women’s screening and preventive behaviours after risk communication. Most women (94.8%) indicated that they adhered to their risk-based mammography screening recommendation. Perceived need for supplemental mammography screening was relatively high (22.9%). High-risk women in particular performed more breast self-examination after risk feedback. Higher self-reported breast cancer risk was associated with supplemental mammography intent (moderate vs. average OR_adj_ 3.88, 95% CI 1.86, 8.07) and increased breast self-examination (high vs. average OR_adj_ 3.83, 95% CI 1.89, 7.77) (Table 3). Having a first degree family history of breast cancer was associated with supplemental mammography intent (OR_adj_ 2.03, 95% CI 1.02, 4.03). This association was also found in our sensitivity analysis of women who accurately reported their counselled breast cancer risk (Supplement 1).

**Table 2:** Women’s organisational preferences and adoption of health behaviours after risk feedback

			**Self-reported breast cancer risk**							
	**All women** ***N*** **= 325**		**Low** ***N*** **= 60**		**Average** ***N*** **= 125**		**Moderate** ***N*** **= 80**		**High** ***N*** **= 60**	
Risk result in letter, n acceptable (%)^a^	251	(77.2)	53	(88.3)	104	(83.2)	61	(76.3)	33	(55.0)
Need for consultation, n yes (%)	99	(30.5)	2	(3.3)	24	(19.2)	28	(35.0)	45	(75.0)
Preferred risk counsellor, n (%)^b^										
General practitioner	49	(49.5)	1	(50.0)	14	(40.0)	11	(30.6)	23	(30.3)
Oncologist	42	(42.4)	1	(50.0)	11	(31.4)	11	(30.6)	19	(25.0)
Geneticist	37	(37.4)	–	–	2	(5.7)	10	(27.7)	25	(32.9)
Nurse	21	(21.2)	–	–	8	(22.9)	4	(11.1)	9	(11.8)
Radiologist	–	–	–	–	–	–	–	–	–	–
Radiographer	–	–	–	–	–	–	–	–	–	–
Use website, n yes (%)	243	(74.8)	41	(68.3)	97	(77.6)	59	(73.8)	46	(76.7)
Screening intent, n yes (%)^c^	308	(94.8)	54	(90.0)	123	(98.4)	78	(97.5)	53	(88.3)
Supplemental mammography intent, n yes (%)^d^	47	(22.9)	n/a		16	(12.8)	31	(38.8)	n/a	
Preferred screening interval low risk										
3-year			34	(56.7)						
4-year			20	(33.3)						
5-year			4	(6.7)						
Don’t know			2	(3.3)						
Preferred screening interval high risk										
6-month									5	(8.3)
1-year									34	(56.7)
18-month									16	(26.6)
2-year									3	(5.0)
3-year									1	(1.7)
Don’t know									1	(1.7)
	**All women** ***N*** **= 325**		**Low risk** ***N*** **= 60**		**Average risk** ***N*** **= 125**		**Moderate risk** ***N*** **= 80**		**High risk** ***N*** **= 60**	
Increased breast self-exam, n yes (%)	124	(38.2)	12	(20.0)	36	(28.8)	39	(48.8)	37	(61.7)
Changed diet (%)										
Yes	77	(23.7)	7	(11.7)	20	(16.0)	23	(28.7)	27	(45.0)
No	69	(21.2)	10	(16.7)	37	(29.6)	12	(15.0)	10	(16.7)
No, not required	179	(55.1)	43	(71.6)	68	(54.4)	45	(56.3)	23	(38.3)
Changed exercise habits, n (%)										
Yes	86	(26.5)	16	(26.7)	28	(22.4)	16	(20.0)	18	(30.0)
No	102	(31.4)	15	(25.0)	48	(38.4)	23	(28.8)	16	(26.7)
No, not required	137	(42.1)	29	(48.3)	49	(39.2)	41	(51.2)	26	(43.3)
Changed alcohol intake, n (%)										
Yes	65	(20.0)	12	(20.0)	22	(17.6)	17	(21.2)	14	(23.3)
No	107	(34.8)	17	(28.3)	47	(37.6)	23	(28.8)	20	(33.3)
No, not required	153	(47.2)	31	(51.7)	56	(44.8)	40	(50.0)	26	(43.4)
Started medication, n yes (%)^e^	46	(50.0)	n/a		n/a		13	(31.7)	33	(66.0)
Willing to consider medication, n yes (%)^f^	16	(33.3)	n/a		n/a		13	(33.3)	3	(33.3)
Tamoxifen preference, n (%)										
Pill	73	(52.1)	n/a		n/a		32	(40.0)	41	(68.4)
Cream	22	(15.7)	n/a		n/a		17	(21.3)	5	(8.3)
No preference	9	(6.4)	n/a		n/a		8	(10.0)	1	(1.7)
Neither	28	(20.1)	n/a		n/a		17	(21.3)	11	(18.3)
Don’t know	8	(5.7)	n/a		n/a		6	(7.4)	2	(3.3)

^a^ High-risk women did not receive their risk result in a letter, but were asked about a hypothetical scenario; ^b^ Percentages based on the number of women who perceived a need for a consultation, women could mark multiple options; ^c^ Based on stratified interval displayed in Fig. 1; ^d^ Based on a 4-year screening interval for low-risk women, and a 3-year screening interval for average and moderate risk women, ^e^ Based on number of women who indicated that tamoxifen was discussed with them for the PROCAS study; ^f^ Based on number of women who were eligible for preventative medication but indicated that it was not discussed with them for the PROCAS study

**Table 3:** Explorative analyses of factors associated with early detection behaviours after risk feedback with tailored screening recommendations

Characteristic	Supplemental mammography intent	Increased breast self-examination
	Multi-adjusted^**a**^	Multi-adjusted^**a**^
	OR^b^ (95% CI)	OR^b^ (95% CI)
Self-reported breast cancer risk		
Low	n/a	0.66 (0.30, 1.45)
Average	Reference	Reference
Moderate	**3.88** (1.86, 8.07)	**2.43** (1.30, 4.53)
High	n/a	**3.83** (1.89, 7.77)
Age (year)^c^	1.03 (0.97, 1.10)	0.98 (0.93, 1.03)
Education		
Lower	Reference	Reference
Higher secondary	1.03 (0.44, 2.39)	0.90 (0.46, 1.74)
Higher vocational	0.73 (0.32, 1.63)	0.54 (0.28, 1.03)
FDR^d^ with breast cancer		
No	Reference	Reference
Yes	**2.03** (1.02, 4.03)	1.10 (0.64, 1.90)
Benign breast disease		
No	Reference	Reference
Yes	1.20 (0.65, 2.23)	1.46 (0.89, 2.41)
Previous breast biopsy		
No	Reference	Reference
Yes	1.56 (0.63, 3.83)	1.45 (0.71, 2.95)
General health^e^	0.99 (0.97, 1.01)	0.99 (0.97, 1.01)
Health anxiety^f^	0.99 (0.92, 1.07)	1.01 (0.95, 1.07)
Health locus of control^g^		
No preference	Reference	Reference
Internal	0.94 (0.31, 2.83)	1.18 (0.49, 2.86)
Chance	1.12 (0.39, 3.20)	1.99 (0.87, 4.57)

^a^ Adjusted for age, education, first degree relative with breast cancer, benign breast disease, general health, breast cancer risk, and health anxiety; ^b^ Odds ratios in bold are significant with *p* < 0.05; ^c^ Age per 1 year increase; ^d^ FDR = first degree relative; ^e^ General health per one point increase; ^f^ Health anxiety per one point increase; ^g^ *n* = 11 people with a physician health locus of control were excluded due to small sample size

### Preventive behaviours after risk communication

High-risk women were more likely than the other risk groups to have changed their diet, exercise and alcohol intake habits (Table 2). More high-risk (66.0%) than moderate risk (31.7%) women started taking risk-reducing medication (tamoxifen or raloxifene), preferring oral to topical medication. Self-reported breast cancer risk was associated with increased adoption of a healthy diet (high vs. average OR_adj_ 4.60, 95% CI 2.03, 10.42), exercise (high vs. average OR_adj_ 2.18, 95% CI 1.05, 4.56), and risk-reducing medication (moderate vs. high OR_adj_ 0.12, 95% CI 0.04, 0.39) (Table 4). Higher age was associated with taking risk-reducing medication (OR_adj_ 1.13, 95% CI 1.01, 1.28). This association was not found in our sensitivity analysis of women who correctly reported their breast cancer risk to be moderate or high (Supplement 1). Higher BMI was associated with the adoption of a healthy diet (OR_adj_ 1.24, 95% CI 1.13, 1.36) and exercise (OR_adj_ 1.10, 95% CI 1.01, 1.19). This association persisted for BMI in the sensitivity analyses (Supplement 1).

**Table 4:** Explorative analyses of factors associated with preventive behaviours after breast cancer risk feedback with tailored prevention recommendations

Characteristic	Started medication	Changed diet	Increased exercise	Limited alcohol intake
	Multi-adjusted^a^	Multi-adjusted^a^	Multi-adjusted^a^	Multi-adjusted^a^
	OR^b^ (95% CI)	OR^b^ (95% CI)	OR^b^ (95% CI)	OR^b^ (95% CI)
Self-reported breast cancer risk				
Low	n/a	0.79^f^ (0.29, 2.16)	1.71 (0.80, 3.66)	1.22^f^ (0.54, 2.77)
Average	n/a	Reference	Reference	Reference
Moderate	**0.12**^f^ (0.04, 0.39)	**2.57** (1.19, 5.55)	0.89 (0.43, 1.85)	1.26 (0.60, 2.64)
High	Reference	**4.60** (2.03, 10.42)	**2.18** (1.05, 4.56)	1.44 (0.64, 3.24)
Age^g^	**1.13** (1.01, 1.28)	0.98 (0.92, 1.05)	1.01 (0.96, 1.07)	1.01 (0.95, 1.07)
Education				
Lower	0.61^f^ (0.15, 2.41)	Reference	Reference	Reference
Higher secondary	2.05 (0.64, 6.62)	1.42 (0.63, 3.22)	1.08 (0.52, 2.23)	1.14 (0.53, 2.49)
Higher vocational	Reference	1.13 (0.50, 2.55)	1.11 (0.55, 2.27)	0.78 (0.36, 1.67)
BMI^h^	0.98 (0.84, 1.14)	**1.24** (1.13, 1.36)	**1.10** (1.01, 1.19)	0.99 (0.91, 1.08)
FDR^c^ breast cancer				
No	Reference	Reference	Reference	Reference
Yes	0.42 (0.13, 1.37)	1.11 (0.57, 2.13)	1.70 (0.93, 3.11)	1.05 (0.56, 1.98)
Benign breast disease				
No	Reference	Reference	Reference	Reference
Yes	0.83 (0.29, 2.37)	0.88 (0.47, 1.64)	0.75 (0.43, 1.30)	1.02 (0.57, 1.83)
Previous breast biopsy				
No	Reference	Reference	Reference	Reference
Yes	1.76 (0.31, 9.89)	1.31 (0.55, 3.16)	0.90 (0.40, 2.00)	2.01 (0.83, 4.91)
Co-morbidity				
0–1	Reference	Reference	Reference	Reference
≥ 2	1.37 (0.48, 3.93)	1.42 (0.75, 2.70)	0.62 (0.35, 1.11)	0.77 (0.42, 1.41)
General health^i^	1.00 (0.95, 1.04)	1.00 (0.98, 1.02)	0.99 (0.97, 1.01)	1.00 (0.98, 1.03)
Life events				
0–1	Reference	Reference	Reference	Reference
≥ 2	0.47 (0.15, 1.41)	0.63 (0.32, 1.22)	0.92 (0.52, 1.64)	0.53 (0.27, 1.06)
Health anxiety^j^	0.88 (0.77, 1.00)	1.04 (0.97, 1.12)	1.01 (0.94, 1.07)	1.00 (0.94, 1.08)
Current medication use^d^				
No	Reference	n/a	n/a	n/a
Yes	1.09 (0.38, 3.18)	n/a	n/a	n/a
Beliefs about medicines^e^				
Harm	0.92 (0.72, 1.17)	n/a	n/a	n/a
Overuse	0.89 (0.74, 1.06)	n/a	n/a	n/a

^a^ Adjusted for age, education, BMI, first degree relative with breast cancer, benign breast disease, general health, breast cancer risk, and health anxiety; ^b^ Odds ratios in bold are significant with *p* < 0.05; ^c^ *FDR* first degree relative; ^d^ Additionally adjusted for beliefs about medicines; ^e^ Additionally adjusted for current medication use; ^f^ Estimate based on a subgroup with fewer than 10 study participants; ^g^ Age per 1 year increase; ^h^ BMI per 1 point increase; ^i^ General health per one point increase; ^j^ Health anxiety per one point increase

### Preferences for organisation of healthcare

Table 2 provides an overview of women’s organisational preferences, stratified by their self-reported breast cancer risk. Most self-reported low, average and moderate risk women found it acceptable to receive their risk result in a letter, with 75% of high-risk women requesting a face-to-face consultation with either a GP or oncologist. Most low-risk women would prefer to maintain their screening interval of 3 years (56.7%) or extend the interval to 4 years (33.3%). Only 26.7% of high-risk women were satisfied with their proposed screening interval of 18 months, with 65% of women preferring a shorter interval. We also asked low and high-risk women their preferred starting age for screening. Most low-risk women (70%) preferred to maintain the current starting age of 50. Most high-risk women would prefer to start screening at age 40 (80%).

## Discussion

Mammography screening uptake after risk communication is high among all risk groups, which is in line with previous findings [3], although the overrepresentation of high-risk women might have identified those most likely to be adherent. Risk communication leads to higher breast cancer awareness as illustrated by women increasing the frequency of breast self-examination. Higher self-reported breast cancer risk and higher BMI were most consistently associated with the adoption of preventive health behaviours.

Women’s satisfaction with their recommended risk-tailored screening interval is uncertain, reporting high perceived need for supplemental mammography outside the national screening programme. High-risk women would prefer annual screening. The majority of modelling studies conclude that annual screening provides the best benefit-to-harm ratio for high-risk women, although definitions of high risk differ across studies [22–24]. Some modelling studies advocate biennial screening because of the substantial increase in false-positive screening test results associated with annual screening [25]. Alternatively, lowering the starting age of screening from 50 to 40 years for high-risk women can also increase screening benefit [25]. This would correspond to high-risk women’s preferred starting age. Moreover, high-risk women with extremely dense breast tissue can benefit from supplemental MRI screening [26]. Most low-risk women wanted to maintain their current screening interval of 3 years (56.7%). This infers that acceptability of decreasing the screening frequency for low-risk women is low, which is in line with previous research [13].

Lifestyle interventions were mostly adopted by women with a higher BMI and women who were at a self-reported increased risk of developing breast cancer. This suggests that risk feedback can motivate women for whom lifestyle interventions are likely to have the greatest benefit. It corresponds to a previous study among PROCAS participants which showed that women with an increased breast cancer risk were significantly more likely to join and remain in two weight loss programmes than low-risk women, and consequently had lost more weight at 12-month follow-up [12]. However, previous research has shown that risk-based lifestyle recommendations do not result in sustained changes in health-related behaviours [8, 9]. Therefore, women’s long-term adherence to the weight loss interventions will have to be evaluated.

Self-reported low-risk women’s apparent disengagement with lifestyle interventions is concerning in light of the general health benefits that can be achieved [12]. There is also a group of women who are still undecided on the uptake of preventive measures. Around 30% of women who say they could benefit from preventive lifestyle interventions have not yet adopted any. Additionally, one third of women eligible for risk-reducing medication based on their self-reported risk were still considering uptake years after receiving their breast cancer risk feedback. A previous focus group study with PROCAS participants provided insights into incentives and barriers to the uptake of preventative measures for breast cancer [13]. Women were sceptical of the link between lifestyle and breast cancer, citing inconsistent messages in the media as the main reason for their scepticism. They also emphasised the difficulty of maintaining a healthy lifestyle. Barriers to the uptake of risk-reducing medication were potential side effects and the perceived daily hassle. Women want to be convinced that the perceived barriers of lifestyle changes and medication weigh up to the breast cancer risk reduction that can be achieved. This underlines the importance of comprehensive information materials and decision aids which outline the benefits and harms of all risk-tailored screening and preventive options.

The discordance between counselled breast cancer risk and self-reported risk may mean that PROCAS participants currently do not receive optimal early detection and preventive care according to risk-based guidance. More than half of high-risk women who would have been eligible for more intensive screening, reported a lower to moderately increased risk. These women may be unaware of the additional screening they can request. In addition, moderate to high-risk women who have reported an average to low breast cancer risk (31.6%) may be unaware that they are eligible for risk-reducing medication in the UK. Recall of health risks is known to be suboptimal [27, 28] and therefore the results of this study are not entirely surprising. Retention of risk and the meaning of test results appears particularly poor when the information is less personally involving, e.g., screen-negative versus screen-positive individuals [27]. People have a tendency to simplify complex risk information, for example by reducing its meaning to either ‘I’m at risk’ or ‘I’m not at risk’, thereby reducing the cognitive effort required to understand complex risk information [29]. PROCAS participants who were informed to be at average or low risk may have remembered that they were not at increased risk. This may have resulted in the terms ‘low’ and ‘average’ being used interchangeably, since neither required changes to their screening policy. Additionally, systematic recall bias in individuals who received the most undesirable, personally threatening test result has been reported before, with individuals recalling lower, i.e., healthier, risk categories than counselled [28]. This appears to be an attempt to reduce the perceived threat of the risk information and thereby any associated distress [30]. PROCAS participants’ risk recall in this study is mostly in line with this, showing bias towards reduced risk. This has been reported before, for example in a study of risk recall among cystic fibrosis carriers whose long-term (3-year) risk recall and understanding was poor and biased towards reduced risk [27]. The discordance between counselled breast cancer risk and self-reported risk can also be a result of risk status altering in the light of new information. Within the 31–52 weeks after risk feedback a woman’s risk factors, e.g., family history, MHT use, or benign breast disease, may have changed, altering her risk perception. Women’s risk recall and understanding could be improved by taking into account women’s likely prior knowledge and by presenting risk more vividly, e.g., with visual images [31].

Successful implementation of risk-based breast cancer screening and prevention relies heavily on women’s participation and adherence to recommended care pathways. If risk-based screening is implemented in practice, routine invitations in line with a woman’s tailored screening interval will be issued, taking some of the responsibility away from women. For successful implementation of risk-based prevention measures, we may have to look outside of the current screening infrastructure to, for example, primary care professionals for assistance. Establishing a chain of responsibility from screening to primary care professionals could aid women’s uptake and adherence to preventive measures. In many European countries, general practitioners (GPs) are optimally positioned to gauge a woman’s preferences and motivation regarding breast cancer prevention. Their knowledge of a woman’s (medical) history and homelife, combined with a relatively high frequency of contact, will enable GPs to effectively monitor women’s progress and wishes.

Two experimental studies are currently investigating whether risk-based screening is at least equally or potentially more effective and efficient than age-based screening. In the US, the Women Informed to Screen Depending on Measures of risk (WISDOM) project was initiated in 2016 [32]. It is a multi-centre preference-tolerant trial that compares annual mammography to a risk-based approach in 40–74 year old women. Four breast cancer risk categories are distinguished, i.e., lowest, average, elevated, and highest risk, with subsequent screening strategies varying from no screening until age 50, biennial screening, annual mammography, and annual mammography with supplemental MRI, respectively. In Europe, the My Personal Breast Screening (MyPEBS) study started recruitment in 2019 [http://mypebs.eu/en/the-project/]. This randomised, open-label, multi-centre study includes women from six different countries, comparing current screening strategies with risk-tailored strategies. Four risk categories with subsequent screening strategies are studied, i.e., low-risk women – mammography at 4-year intervals, moderate-risk women – mammography at 2-year intervals, high risk women – annual mammography, and very high-risk women – annual mammography and supplemental MRI until aged 60. These clinical trials will provide important information on the cost-effectiveness of risk-based screening. Additionally, two large feasibility studies are currently underway, i.e., the BC-predict study in the UK [33] and the Perspective I&I study in Canada [34], exploring implementation issues such as uptake rates, and acceptability to participating women and healthcare professionals. If implementation proves warranted and feasible, the present study offers valuable insights into the role of risk communication in women’s uptake of screening and prevention recommendations and women’s organisational preferences.

### Strengths and limitations

This is one of the first studies to explore factors associated with uptake of early detection and preventive behaviours after breast cancer risk communication in the screening setting. Previous studies have primarily focused on intent. There are, however, some limitations that need to be considered when interpreting the results. We had a relatively low response rate of 11.6%. We tried to maximise response by sending women personally addressed study invitations. However, PROCAS participants are approached for a number of different follow-up studies. Therefore, they may have already been approached for other studies in the past. This survey study also required a considerable time investment of 20–30 min which may have put some women off participation. Although we aimed for equal representation of all breast cancer risk groups, women at increased risk (as identified by the PROCAS study) were overrepresented in the study. Moreover, the prevalence of a positive first-degree breast cancer family history among study participants was very high (42.5%) compared to the general population (6.7%) [35] and PROCAS study participants as a whole (11.8%). This makes the generalisability of our findings to the general screening population uncertain. We performed logistic regression analysis, since we did not anticipate differences in ‘time since risk feedback’ to have a significant impact on uptake of screening and prevention recommendations. However, with our results showing relatively poor risk recall, time since risk feedback may be an interesting factor for future research, with a focus on the period shortly after feedback.

## Conclusions

Breast cancer risk communication predicts the uptake of personalised screening and prevention recommendations. Having a first-degree family history of breast cancer was most consistently associated with the uptake of breast care behaviours, whereas having a high BMI was the biggest motivator for lifestyle alterations. Tailored screening can more closely correspond to women’s organisational preferences by further shortening the interval and moving the starting age forward for women at increased risk.

## Supplementary Information

**Additional file 1.**

**Additional file 2.**

**Additional file 3.**

**Additional file 4.**

**Additional file 5.**

## Data Availability

The datasets used and/or analysed during the current study are available from the corresponding author on reasonable request.

## Abbreviations

BMI: Body mass index
NHSBSP: National Health Service Breast Screening Programme
PROCAS: Predicting Risk of Cancer at Screening
TC: Tyrer-Cuzick
UK: United Kingdom

## References

[1] NHS Digital. Breast Screening Programme, England, 2016–17. 2018. Available from: https://digital.nhs.uk/catalogue/PUB30195 (Retrieved 20 August 2020).

[2] Marmot M, Altman G, Cameron DA, Dewar JA, Thompson SG, Wilcox M, Independent UK Panel on Breast Cancer Screening (2013). Independent UK panel on breast cancer screening replies to Michael Baum. BMJ.

[3] Evans DGR, Donnelly LS, Harkness EF, Astley SM, Stavrinos P, Dawe S (2016). Breast cancer risk feedback to women in the UKNHS breast screening population. Br J Cancer.

[4] Tyrer J, Duffy SW, Cuzick J (2004). A breast cancer prediction model incorporating familial and personal risk factors. Stat Med.

[5] Nelson HD, Fu R, Zakher B, Pappas M, McDonagh M (2019). Medication use for the risk reduction of primary breast cancer in women: updated evidence report and systematic review for the US preventive services task force. JAMA..

[6] Kushi LH, Doyle C, McCullough M, Rock CL, Demark-Wahnefried W, Bandera EV (2012). American Cancer Society guidelines on nutrition and physical activity for cancer prevention: reducing the risk of cancer with healthy food choices and physical activity. CA Cancer J Clin.

[7] French DP, Southworth J, Howell A, Harvie M, Stavrinos P, Watterson D (2018). Psychological impact of providing women with personalised 10-year breast cancer risk estimates. Br J Cancer.

[8] Hollands GJ, French DP, Griffin SJ, Prevost AT, Sutton S, King S, Marteau TM (2016). The impact of communicating genetic risks of disease on risk-reducing health behaviour: systematic review with meta-analysis. BMJ.

[9] French DP, Cameron E, Benton JS, Deaton C, Harvie M (2017). Can communicating personalised disease risk promote healthy behaviour change? A systematic review of systematic reviews. Ann Behav Med.

[10] Sheeran P, Harris PR, Epton T (2014). Does heightening risk appraisals change people’s intentions and behavior? A meta-analysis of experimental studies. Psychol Bull.

[11] Atkinson TM, Salz T, Touza KK, Li Y, Hay JL (2015). Does colorectal cancer risk perception predict screening behavior? A systematic review and meta-analysis. J Behav Med.

[12] Harvie M, Pegington M, French D, Cooper G, McDiarmid S, Howell A (2019). Breast cancer risk status influences uptake, retention and efficacy of a weight loss programme amongst breast cancer screening attendees: two randomised controlled feasibility trials. BMC Cancer.

[13] Rainey L, Jervaeus A, Donnelly LS, Evans DG, Hammarström M, Hall P (2019). Women's perceptions of personalized risk-based breast cancer screening and prevention: an international focus group study. Psychooncol..

[14] The EuroQol Group (1990). EuroQol-a new facility for the measurement of health-related quality of life. Health Policy.

[15] Salkovskis PM, Rimes KA, Warwick HM, Clark DM (2002). The health anxiety inventory: development and validation of scales for the measurement of health anxiety and hypochondriasis. Psychol Med.

[16] te Poel F, Hartmann T, Baumgartner SE, Tanis M (2017). A psychometric evaluation of the Dutch short health anxiety inventory in the general population. Psychol Assess.

[17] Wallston KA, Wallston BS (1978). Development of the multidimensional health locus of control (MHLC) scales. Health Educ Monographs.

[18] Wallston KA (2005). The validity of the multidimensional health locus of control scales. J Health Psychol.

[19] Holmes TH, Rahe RH (1967). The social readjustment rating scale. J Psychosom Res.

[20] Horne R, Weinman J, Hankins M (1999). The beliefs about medicines questionnaire: the development and evaluation of a new method for assessing the cognitive representation of medication. Psychol Health.

[21] Rainey L, van der Waal D, Wengström Y, Jervaeus A, Broeders MJ (2018). Women’s perceptions of the adoption of personalised risk-based breast cancer screening and primary prevention: a systematic review. Acta Oncol.

[22] Gray E, Donten A, Karssemeijer N, van Gils C, Evans DG, Astley S, Payne K (2017). Evaluation of a stratified national breast screening program in the United Kingdom: an early model-based cost-effectiveness analysis. Value Health.

[23] Trentham-Dietz A, Kerlikowske K, Stout NK, Miglioretti DL, Schechter CB, Ergun MA (2016). Tailoring breast cancer screening intervals by breast density and risk for women aged 50 years or older: collaborative modeling of screening outcomes. Ann Intern Med.

[24] Vilaprinyo E, Forne C, Carles M, Sala M, Pla R, Castells X, et al. Cost-effectiveness and harm-benefit analyses of risk-based screening strategies for breast cancer. PLoS One. 2014;9(2):e86858. 10.1371/journal.pone.0086858 PMC3911927 24498285

[25] Sankatsing VD, van Ravesteyn NT, Heijnsdijk EA, Broeders MJ, de Koning HJ (2020). Risk stratification in breast cancer screening: cost-effectiveness and harm-benefit ratios for low-risk and high-risk women. Int J Cancer.

[26] Bakker MF, de Lange SV, Pijnappel RM, Mann RM, Peeters PH, Monninkhof EM (2019). Supplemental MRI screening for women with extremely dense breast tissue. NEJM..

[27] Axworthy D, Marteau TM, Brock DJH, Bobrow M, UK Cystic Fibrosis Follow-up Study (1996). Psychological impact of population-based carrier testing for cystic fibrosis: 3-year follow-up. Lancet..

[28] Croyle RT, Loftus EF, Barger SD, Sun YC, Hart M, Gettig J (2006). How well do people recall risk factor test results? Accuracy and bias among cholesterol screening participants. Health Psychol.

[29] Brainerd CJ, Reyna VF. Fuzzy-trace theory: dual processes in memory, reasoning, and cognitive neuroscience. Adv Child Dev Behav. 2001;28:41–100. 10.1016/s0065-2407(02)80062-3 11605365

[30] Croyle RT, Jemmot JB, Skelton JA, Croyle RT (1991). Psychological reactions to risk factor testing. Mental representation in health and illness.

[31] French DP & Marteau TM. In Llewellyn, C., Ayers, S., McManus, C., Newman, S. P., Petrie, K., Revenson, T., & Weinman, J. (Eds.). Cambridge handbook of psychology, health and medicine. New York: Cambridge University Press 2019.

[32] Esserman LJ (2017). The WISDOM study: breaking the deadlock in the breast cancer screening debate. NPJ. Breast Cancer.

[33] French DP, Astley S, Brentnall AR (2020). What are the benefits and harms of risk stratified screening as part of the NHS breast screening Programme? Study protocol for a multi-site non-randomised comparison of BC-predict versus usual screening (NCT04359420). BMC Cancer.

[34] Brooks JD, Nabi HH, Andrulis IL, Antoniou AC, Chiquette J, Després P (2021). Personalized risk assessment for prevention and early detection of breast Cancer: integration and implementation (PERSPECTIVE I&I). J Pers Med.

[35] Jacobi CE, Jonker MA, Nagelkerke NJD, Van Houwelingen JC, De Bock GH (2003). Prevalence of family histories of breast cancer in the general population and the incidence of related seeking of health care. J Med Gen.

