# Supplementary material for: Women’s health behaviour change after receiving breast cancer risk estimates with tailored screening and prevention recommendations
Source: BMC Cancer. 2022 Jan 16;22:69. doi: 10.1186/s12885-022-09174-3 (PMC8761310; doi:10.1186/s12885-022-09174-3)
Supplement: Supplementary file 1 — Additional file 1. [file 12885_2022_9174_MOESM1_ESM.docx]

**Supplement 1.** Sensitivity analyses

**Supplement 1a.** Sensitivity analysis of participants who correctly reported their counselled breast cancer risk.

**Table 1.** Organisational preferences and health behaviour adoption after risk feedback of women who correctly reported their counselled breast cancer risk

|  |  | | **Counselled breast cancer risk** | | | | | | | |
| --- | --- | --- | --- | --- | --- | --- | --- | --- | --- | --- |
|  | **All women**  **N=172** | | **Low**  **N=30** | | **Average**  **N=49** | | **Moderate**  **N=42** | | **High**  **N=51** | |
| Risk result in letter, n acceptable (%)^a^ | 127 | (73.8) | 27 | (90.0) | 40 | (81.6) | 33 | (78.6) | 27 | (52.9) |
| Need for consultation, n yes (%) | 55 | (32.0) | - | (-) | 6 | (12.2) | 11 | (26.2) | 38 | (74.5) |
| Preferred risk counsellor, n (%)^b^ |  |  |  |  |  |  |  |  |  |  |
| Oncologist | 27 | (49.1) |  | n/a | 4 | (66.7) | 5 | (45.6) | 18 | (47.4) |
| Geneticist | 25 | (45.5) |  | n/a | - | (-) | 4 | (36.4) | 21 | (55.3) |
| General practitioner | 23 | (41.8) |  | n/a | 3 | (50.0) | 3 | (27.3) | 17 | (44.7) |
| Nurse | 10 | (18.2) |  | n/a | 2 | (33.3) | 2 | (18.2) | 6 | (15.8) |
| Radiologist | - | (-) |  | n/a | - | (-) | - | (-) | - | (-) |
| Radiographer | - | (-) |  | n/a | - | (-) | - | (-) | - | (-) |
| Use website, n yes (%) | 127 | (73.8) | 18 | (60.0) | 39 | (79.6) | 30 | (71.4) | 40 | (78.4) |
| Screening intent, n yes (%)^c^ | 160 | (93.0) | 25 | (83.3) | 48 | (98.0) | 41 | (97.6) | 46 | (90.0) |
| Supplemental mammography, n yes (%)^c^ | 19 | (20.9) |  | n/a | 3 | (6.1) | 16 | (38.1) | n/a | |
| Preferred screening interval low risk |  |  |  |  |  |  |  |  |  |  |
| 3-year |  |  | 16 | (53.3) |  |  |  |  |  |  |
| 4-year |  |  | 9 | (30.0) |  |  |  |  |  |  |
| 5-year |  |  | 3 | (10.0) |  |  |  |  |  |  |
| Don’t know |  |  | 2 | (6.7) |  |  |  |  |  |  |
| Preferred screening interval high risk |  |  |  |  |  |  |  |  |  |  |
| 6-month |  |  |  |  |  |  |  |  | 5 | (9.8) |
| 1-year |  |  |  |  |  |  |  |  | 28 | (54.9) |
| 18-month |  |  |  |  |  |  |  |  | 14 | (27.5) |
| 2-year |  |  |  |  |  |  |  |  | 2 | (3.9) |
| 3-year |  |  |  |  |  |  |  |  | 1 | (2.0) |
| Don’t know |  |  |  |  |  |  |  |  | 1 | (2.0) |

**Table 1**. *Continued*

|  | **All women**  **N=172** | | **Low risk**  **N=30** | | **Average risk**  **N=49** | | **Moderate risk**  **N=42** | | **High risk**  **N=51** | |
| --- | --- | --- | --- | --- | --- | --- | --- | --- | --- | --- |
| Increased breast self-exam, n yes (%) | 65 | (37.8) | 4 | (13.3) | 9 | (18.4) | 20 | (47.6) | 32 | (62.7) |
| Changed diet (%) |  |  |  |  |  |  |  |  |  |  |
| Yes | 46 | (26.7) | 3 | (10.0) | 7 | (14.3) | 12 | (28.6) | 24 | (47.1) |
| No | 37 | (21.5) | 7 | (23.3) | 13 | (26.5) | 8 | (19.0) | 9 | (17.6) |
| No, not required | 89 | (51.7) | 20 | (66.7) | 29 | (59.2) | 22 | (52.4) | 18 | (35.3) |
| Changed exercise habits, n (%) |  |  |  |  |  |  |  |  |  |  |
| Yes | 49 | (28.5) | 7 | (23.3) | 10 | (20.4) | 8 | (19.0) | 24 | (47.1) |
| No | 49 | (28.5) | 8 | (26.7) | 20 | (40.8) | 11 | (26.2) | 10 | (19.6) |
| No, not required | 74 | (43.0) | 15 | (50.0) | 19 | (38.8) | 23 | (54.8) | 17 | (33.3) |
| Changed alcohol intake, n (%) |  |  |  |  |  |  |  |  |  |  |
| Yes | 37 | (21.5) | 5 | (16.7) | 8 | (16.3) | 10 | (23.8) | 14 | (27.5) |
| No | 62 | (36.0) | 9 | (30.0) | 24 | (49.0) | 11 | (26.2) | 18 | (35.3) |
| No, not required | 73 | (42.4) | 16 | (53.3) | 17 | (34.7) | 21 | (50.0) | 19 | (37.3) |
| Started medication, n yes (%)^d^ | 33 | (55.9) |  |  |  |  | 4 | (26.7) | 29 | (65.9) |
| Tamoxifen preference, n (%) |  |  |  |  |  |  |  |  |  |  |
| Pill | 48 | (51.6) |  |  |  |  | 11 | (26.2) | 37 | (72.5) |
| Cream | 15 | (16.1) |  |  |  |  | 10 | (23.8) | 5 | (9.8) |
| No preference | 19 | (20.4) |  |  |  |  | 11 | (26.2) | 8 | (15.7) |
| Neither | 6 | (6.5) |  |  |  |  | 5 | (11.9) | 1 | (2.0) |
| Don’t know | 5 | (5.4) |  |  |  |  | 5 | (11.9) | - | (-) |

^a^ High-risk women did not receive their risk result in a letter, but were asked about a hypothetical scenario; ^b^ Percentages based on the number of women who perceived a need for a consultation, women could mark multiple options; ^c^ Based on stratified interval displayed in Fig 1;  ^d^ Based on women who indicated that tamoxifen was presented to them as a preventative option by the PROCAS study, i.e. total n=59, moderate risk n=15, high risk n=44

**Table 2**. Explorative analyses of factors associated with breast care after risk feedback and provision of tailored recommendations on subset of women who correctly reported their breast cancer risk

| **Characteristic^g^** | **Supplemental mammography intent** | | **Increased breast self-examination** | |
| --- | --- | --- | --- | --- |
|  | **Unadjusted** | **Multi-adjusted^a^** | **Unadjusted** | **Multi-adjusted^a^** |
|  | OR (95% CI) | OR (95% CI) | OR (95% CI) | OR (95% CI) |
| Age (year)^c^ | **1.10** (1.01, 1.21) | 1.12 (1.00, 1.25) | 0.97 (0.91, 1.03) | 1.00 (0.92, 1.07) |
| FDR^e^ with breast cancer |  |  |  |  |
| No | Reference | Reference | Reference | Reference |
| Yes | **5.79** (2.23, 15.04) | **8.10** (2.04, 32.20) | **2.86** (1.51, 5.44) | 0.89 (0.36, 2.19) |
| Benign breast disease |  |  |  |  |
| No | Reference | Reference | Reference | Reference |
| Yes | 1.62 (0.67, 3.93) | 0.71 (0.22, 2.33) | 1.60 (0.84, 3.04) | 0.99 (0.45, 2.18) |
| General health^d^ | 0.99 (0.97, 1.02) | 0.99 (0.96, 1.03) | 0.99 (0.97, 1.01) | 0.99 (0.96, 1.01) |
| Health anxiety^f^ | 0.99 (0.90, 1.10) | 1.03 (0.91, 1.18) | **1.08** (1.00, 1.15) | 1.05 (0.96, 1.14) |

^a^ Adjusted for age, education, first degree relative with breast cancer, benign breast disease, general health, breast cancer risk, and health anxiety; ^b^ Odds ratios in bold are significant with p<0.05; ^c^ Age per 1 year increase; ^d^ General health per one point increase ^e^ FDR = first degree relative; ^f^ Health anxiety per one point increase; ^g^ Characteristics with subgroups of n ≥10 are included in these analyses

**Table 3**. Explorative analyses of factors associated with preventive behaviours after risk feedback and provision of tailored recommendations on subset of women who correctly reported their breast cancer risk

| **Characteristic^h^** | **Changed diet** | | **Increased exercise** | | **Limited alcohol intake** | | **Started medication** | |
| --- | --- | --- | --- | --- | --- | --- | --- | --- |
|  | **Unadjusted** | **Multi-adjusted**^a^ | **Unadjusted** | **Multi-adjusted**^a^ | **Unadjusted** | **Multi-adjusted**^a^ | **Unadjusted** | **Multi-adjusted**^a^ |
|  | OR (95% CI) | OR (95% CI) | OR (95% CI) | OR (95% CI) | OR (95% CI) | OR (95% CI) | OR (95% CI) | OR (95% CI) |
| Age^c^ | 0.96 (0.90, 1.03) | 0.97 (0.89, 1.06) | 1.02 (0.95, 1.09) | 1.03 (0.95, 1.11) | 0.98 (0.91, 1.06) | 0.99 (0.91, 1.08) | 1.01 (0.90, 1.14) | 1.01 (0.88, 1.16) |
| BMI^d^ | **1.21** (1.09, 1.34) | **1.23** (1.09, 1.40) | **1.16** (1.05, 1.28) | **1.15** (1.03, 1.28) | 0.95 (0.85, 1.06) | 0.95 (0.84, 1.08) | 0.98 (0.85, 1.13) | 0.97 (0.81, 1.16) |
| General health | **0.97** (0.95, 1.00) | 0.99 (0.96, 1.02) | 0.98 (0.96, 1.00) | 0.99 (0.96, 1.02) | 1.00 (0.98, 1.03) | 1.01 (0.98, 1.04) | 1.01 (0.97, 1.05) | 0.99 (0.94, 1.04) |
| FDR^e^ breast cancer |  |  |  |  |  |  |  |  |
| No | Reference | Reference | Reference | Reference | Reference | Reference | Reference | Reference |
| Yes | **2.80** (1.40, 5.59) | 1.08 (0.38, 3.04) | **2.64** (1.34, 5.21) | 1.97 (0.73, 5.34) | 1.12 (0.53, 2.36) | 0.72 (0.27, 1.95) | 1.41 (0.46, 4.30) | 0.74 (0.17, 3.19) |
| Benign breast disease |  |  |  |  |  |  |  |  |
| No | Reference | Reference | Reference | Reference | Reference | Reference | Reference | Reference |
| Yes | 1.10 (0.54, 2.23) | 0.61 (0.24, 1.56) | 0.81 (0.40, 1.66) | 0.62 (0.26, 1.45) | 1.09 (0.51, 2.35) | 0.87 (0.37, 2.03) | 1.33 (0.47, 3.80) | 1.61 (0.46, 5.64) |
| Health anxiety^f^ | **1.11** (1.03, 1.20) | 1.09 (0.99, 1.20) | 1.04 (0.96, 1.12) | 1.02 (0.93, 1.11) | 1.04 (0.96, 1.13) | 1.05 (0.96, 1.16) | 0.93 (0.84, 1.04) | 0.88 (0.76, 1.03) |
| Beliefs about medicines^g^ |  |  |  |  |  |  |  |  |
| Harms | n/a | n/a | n/a | n/a | n/a | n/a | 0.98 (0.76, 1.25) | 1.09 (0.79, 1.50) |
| Overuse | n/a | n/a | n/a | n/a | n/a | n/a | 0.86 (0.70, 1.06) | 0.86 (0.67, 1.11) |

^a^ Adjusted for age, education, BMI, first degree relative with breast cancer, benign breast disease, general health, breast cancer risk, and health anxiety; ^b^ Odds ratios in bold are significant with p<0.05; ^c^ Age per 1 year increase; ^d^ BMI per 1 point increase; ^e^ FDR = first degree relative; ^f^ Health anxiety per one point increase; ^g^ Additionally adjusted for current medication use; ^h^ Characteristics with subgroups of n ≥10 are included in these analyses

**Supplement 1b.** Sensitivity analyses of participants who incorrectly reported their counselled breast cancer risk.

**Table 1.** Organisational preferences and health behaviour adoption after risk feedback of women who incorrectly reported their counselled breast cancer risk

|  |  | | **Counselled breast cancer risk** | | | | | | | |
| --- | --- | --- | --- | --- | --- | --- | --- | --- | --- | --- |
|  | **All women**  **N=128** | | **Low**  **N=30** | | **Average**  **N=59** | | **Moderate**  **N=34** | | **High**  **N=5** | |
| Risk result in letter, n acceptable (%)^a^ | 105 | (82.0) | 26 | (86.7) | 50 | (84.7) | 26 | (76.5) | 3 | (60.0) |
| Need for consultation, n yes (%) | 38 | (29.7) | 2 | (6.7) | 15 | (25.4) | 16 | (47.1) | 5 | (100) |
| Preferred risk counsellor, n (%)^b^ |  |  |  |  |  |  |  |  |  |  |
| Oncologist | 12 | (9.4) | 1 | (3.3) | 7 | (11.9) | 4 | (11.8) | 0 | (-) |
| Geneticist | 11 | (8.6) | 0 | (-) | 2 | (3.4) | 6 | (17.6) | 3 | (60.0) |
| General practitioner | 23 | (18.0) | 1 | (3.3) | 10 | (16.9) | 8 | (23.5) | 4 | (80.0) |
| Nurse | 10 | (7.8) | 0 | (-) | 6 | (10.2) | 2 | (5.9) | 2 | (40.0) |
| Radiologist | 0 | (-) | 0 | (-) | 0 | (-) | 0 | (-) | 0 | (-) |
| Radiographer | 0 | (-) | 0 | (-) | 0 | (-) | 0 | (-) | 0 | (-) |
| Use website, n yes (%) | 102 | (79.7) | 23 | (76.7) | 47 | (79.7) | 28 | (82.4) | 4 | (80.0) |
| Screening intent, n yes (%)^c^ | 125 | (97.7) | 29 | (96.7) | 59 | (100) | 33 | (97.1) | 4 | (80.0) |
| Supplemental mammography, n yes (%)^c^ | 25 | (26.9) | n/a | | 11 | (18.6) | 14 | (41.2) |  | n/a |
| Preferred screening interval low risk |  |  |  |  |  |  |  |  |  |  |
| 3-year |  |  |  | 17 |  |  |  |  |  |  |
| 4-year |  |  |  | 11 |  |  |  |  |  |  |
| 5-year |  |  |  | 0 |  |  |  |  |  |  |
| Don’t know |  |  |  | 2 |  |  |  |  |  |  |
| Preferred screening interval high risk |  |  |  |  |  |  |  |  |  |  |
| 6-month |  |  |  |  |  |  |  |  | 0 |  |
| 1-year |  |  |  |  |  |  |  |  | 3 | (60.0) |
| 18-month |  |  |  |  |  |  |  |  | 2 | (40.0) |
| 2-year |  |  |  |  |  |  |  |  | 0 |  |
| 3-year |  |  |  |  |  |  |  |  | 0 |  |
| Don’t know |  |  |  |  |  |  |  |  | 0 |  |

**Table 1**. *Continued*

|  | **All women**  **N=128** | | **Low risk**  **N=30** | | **Average risk**  **N=59** | | **Moderate risk**  **N=34** | | **High risk**  **N=5** | |
| --- | --- | --- | --- | --- | --- | --- | --- | --- | --- | --- |
| Increased breast self-exam, n yes (%) | 47 | (36.7) | 8 | (26.7) | 20 | (33.9) | 17 | (50.0) | 2 | (40.0) |
| Changed diet (%) |  |  |  |  |  |  |  |  |  |  |
| Yes | 26 | (20.3) | 4 | (13.3) | 9 | (15.3) | 11 | (32.4) | 2 | (40.0) |
| No | 25 | (19.5) | 3 | (10.0) | 19 | (32.2) | 2 | (5.9) | 1 | (20.0) |
| No, not required | 76 | (59.4) | 23 | (76.7) | 31 | (52.5) | 21 | (61.8) | 1 | (20.0) |
| Changed exercise habits, n (%) |  |  |  |  |  |  |  |  |  |  |
| Yes | 32 | (25.0) | 10 | (33.3) | 14 | (23.7) | 8 | (23.5) | 0 | (-) |
| No | 45 | (35.2) | 6 | (20.0) | 25 | (42.4) | 10 | (29.4) | 5 | (100) |
| No, not required | 50 | (39.1) | 14 | (46.7) | 20 | (33.9) | 16 | (47.1) | 0 | (-) |
| Changed alcohol intake, n (%) |  |  |  |  |  |  |  |  |  |  |
| Yes | 25 | (19.5) | 7 | (23.3) | 12 | (20.3) | 6 | (17.6) | 0 | (-) |
| No | 40 | (31.3) | 8 | (26.7) | 19 | (32.2) | 11 | (32.4) | 2 | (40.0) |
| No, not required | 63 | (49.2) | 15 | (50.0) | 28 | (47.5) | 17 | (50.0) | 3 | (60.0) |
| Started medication, n yes (%)^d^ | 11 | (37.9) | n/a | n/a | n/a | n/a | 8 | (23.5) | 3 | (60.0) |
| Tamoxifen preference, n (%) |  |  |  |  |  |  |  |  |  |  |
| Pill | 21 | (55.3) | n/a | n/a | n/a | n/a | 19 | (55.9) | 2 | (40.0) |
| Cream | 6 | (15.8) | n/a | n/a | n/a | n/a | 6 | (17.6) | 0 | (-) |
| No preference | 7 | (7.9) | n/a | n/a | n/a | n/a | 3 | (8.8) | 2 | (40.0) |
| Neither | 3 | (18.4) | n/a | n/a | n/a | n/a | 5 | (14.7) | 0 | (-) |
| Don’t know | 1 | (2.6) | n/a | n/a | n/a | n/a | 1 | (2.9) | 1 | (20.0) |

^a^ High-risk women did not receive their risk result in a letter, but were asked about a hypothetical scenario; ^b^ Percentages based on the number of women who perceived a need for a consultation, women could mark multiple options; ^c^ Based on stratified interval displayed in Fig 1;  ^d^ Based on women who indicated that tamoxifen was presented to them as a preventative option by the PROCAS study, i.e. total n=30, moderate risk n=25, high risk n=5

**Table 2**. Explorative analyses of factors associated with breast care after risk feedback and provision of tailored recommendations on subset of women who incorrectly reported their counselled breast cancer risk

| **Characteristic** | **Supplemental mammography intent** | | **Increased breast self-examination** | |
| --- | --- | --- | --- | --- |
|  | **Unadjusted** | **Multi-adjusted^a^** | **Unadjusted** | **Multi-adjusted^a^** |
|  | OR (95% CI) | OR (95% CI) | OR (95% CI) | OR (95% CI) |
| Age (year)^c^ | 0.97 (0.89, 1.06) | 0.98 (0.88, 1.09) | 0.99 (0.91, 1.07) | 1.01 (0.92, 1.11) |
| FDR^d^ with breast cancer |  |  |  |  |
| No | Reference | Reference | Reference | Reference |
| Yes | 0.82 (0.38, 1.78) | 1.05 (0.33, 3.32) | 1.32 (0.64, 2.72) | 0.58 (0.21, 1.61) |
| Benign breast disease |  |  |  |  |
| No | Reference | Reference | Reference | Reference |
| Yes | 0.79 (0.35, 1.78) | 0.72 (0.27, 1.91) | 1.16 (0.55, 2.46) | 0.80 (0.33, 1.95) |
| General health^e^ | 1.00 (0.97, 1.02) | 0.99 (0.96, 1.03) | 1.00 (0.97, 1.02) | 0.99 (0.96, 1.03) |
| Health anxiety^f^ | 0.99 (0.91, 1.08) | 1.01 (0.90, 1.13) | 0.98 (0.91, 1.05) | 0.97 (0.86, 1.08) |

^a^ Adjusted for age, education, first degree relative with breast cancer, benign breast disease, general health, breast cancer risk, and health anxiety; ^b^ Odds ratios in bold are significant with p<0.05; ^c^ Age per 1 year increase; ^d^ FDR = first degree relative; ^e^ General health per one point increase; ^f^ Health anxiety per one point increase

**Table 3**. Explorative analyses of factors associated with preventive behaviours after risk feedback and provision of tailored recommendations on subset of women who incorrectly reported their breast cancer risk

| **Characteristic** | **Changed diet** | | **Increased exercise** | | **Limited alcohol intake** | |
| --- | --- | --- | --- | --- | --- | --- |
|  | **Unadjusted** | **Multi-adjusted**^a^ | **Unadjusted** | **Multi-adjusted**^a^ | **Unadjusted** | **Multi-adjusted**^a^ |
|  | OR (95% CI) | OR (95% CI) | OR (95% CI) | OR (95% CI) | OR (95% CI) | OR (95% CI) |
| Age^d^ | 0.99 (0.89, 1.09) | 0.94 (0.81, 1.09) | 0.97 (0.88, 1.06) | 0.99 (0.89, 1.11) | 1.04 (0.94, 1.15) | 1.01 (0.90, 1.13) |
| BMI^e^ | **1.25^b^ (1.07, 1.46)** | **1.56 (1.16, 2.09)** | 1.08 (0.95, 1.23) | 1.13 (0.96, 1.32) | 1.09 (0.95, 1.25) | 1.06 (0.89, 1.25) |
| General health^f^ | 1.00 (0.97, 1.03) | 1.02 (0.97, 1.08) | 1.00 (0.97, 1.03) | 1.01 (0.97, 1.05) | 1.00 (0.97, 1.03) | 0.99 (0.95, 1.03) |
| FDR^c^ breast cancer |  |  |  |  |  |  |
| No | Reference | Reference | Reference | Reference | Reference | Reference |
| Yes | 1.26 (0.53, 3.01) | 0.30 (0.05, 1.75) | 0.85 (0.38, 1.89) | 1.50 (0.44, 5.09) | 0.78 (0.32, 1.86) | 0.74 (0.21, 2.60) |
| Benign breast disease |  |  |  |  |  |  |
| No | Reference | Reference | Reference | Reference | Reference | Reference |
| Yes | 0.99 (0.40, 2.46) | 1.47 (0.38, 5.69) | 1.01 (0.44, 2.30) | 1.34 (0.49, 3.64) | 0.96 (0.37, 2.44) | 1.45 (0.48, 4.35) |
| Health anxiety^g^ | 1.04 (0.95, 1.13) | 1.00 (0.81, 1.23) | 1.00 (0.92, 1.09) | 1.09 (0.94, 1.26) | 0.95 (0.86, 1.05) | 1.00 (0.85, 1.17) |

Note: The outcome ‘started medication’ could not be assessed due to small sample sizes (n<10) ^a^ Adjusted for age, education, BMI, first degree relative with breast cancer, benign breast disease, general health, breast cancer risk, and health anxiety; ^b^ Odds ratios in bold are significant with p<0.05; ^c^ FDR = first degree relative; ^d^ Age per 1 year increase; ^e^ BMI per 1 point increase; ^f^ General health per one point increase; ^g^ Health anxiety per one point increase
