# Supplementary material for: Women’s health behaviour change after receiving breast cancer risk estimates with tailored screening and prevention recommendations
Source: BMC Cancer. 2022 Jan 16;22:69. doi: 10.1186/s12885-022-09174-3 (PMC8761310; doi:10.1186/s12885-022-09174-3)
Supplement: Supplementary file 2 — Additional file 2. [file 12885_2022_9174_MOESM2_ESM.docx]

**Supplement 2.** General characteristics of all participants, and stratified by their counselled breast cancer risk

|  |  | | **Counselled breast cancer risk*** | | | | | | | |
| --- | --- | --- | --- | --- | --- | --- | --- | --- | --- | --- |
|  | **All women**  **N=325** | | **Low risk**  **N=48** | | **Average risk**  **N=71** | | **Moderate risk**  **N= 80** | | **High risk**  **N=107** | |
| Age (years), mean (SD)^a^ | 61.3 | (4.9) | 61.9 | (5.1) | 61.4 | (4.9) | 61.5 | (5.2) | 60.9 | (4.6) |
| Education level, n (%)^b^ |  |  |  |  |  |  |  |  |  |  |
| Lower education | 68 | (20.9) | 11 | (22.9) | 12 | (16.9) | 18 | (22.5) | 25 | (23.4) |
| Higher secondary education | 98 | (30.2) | 15 | (31.3) | 20 | (28.2) | 25 | (31.3) | 31 | (29.0) |
| Higher vocational qualification | 131 | (40.3) | 14 | (29.2) | 34 | (47.9) | 32 | (40.0) | 44 | (41.1) |
| Marital status, n living with partner (%) | 256 | (78.8) | 43 | (89.6) | 59 | (83.1) | 61 | (76.3) | 79 | (73.8) |
| First-degree family history breast cancer, n yes (%) | 138 | (42.5) | 2 | (4.2) | 6 | (8.5) | 38 | (47.5) | 84 | (78.5) |
| Body mass index (kg/m^2^), mean (SD)^c^ | 24.9 | (3.5) | 24.5 | (3.4) | 24.7 | (3.3) | 25.3 | (3.6) | 24.7 | (3.6) |
| Medical condition, n ≥ 2 diagnosed (%) | 164 | (50.5) | 24 | (78.9) | 37 | (52.1) | 41 | (51.2) | 47 | (43.9) |
| Current medication use, n yes (%) | 144 | (44.3) | 21 | (43.8) | 38 | (53.5) | 31 | (38.8) | 39 | (36.4) |
| Current MHT^d^ use, n yes (%) | 26 | (8.0) | 9 | (18.8) | 8 | (11.3) | 6 | (7.5) | 2 | (1.9) |
| Benign breast disease, n yes (%)^e^ | 121 | (37.2) | 17 | (35.4) | 14 | (19.7) | 37 | (46.3) | 44 | (41.1) |
| Previous breast biopsy, n yes (%)^f^ | 81 | (24.9) | 6 | (12.5) | 12 | (16.9) | 23 | (28.7) | 34 | (31.8) |
| General health score, mean (SD) | 82.7 | (14.6) | 82.7 | (18.5) | 83.7 | (13.3) | 84.9 | (12.6) | 82.4 | (13.6) |
| Belief in medicines, mean (SD) |  |  |  |  |  |  |  |  |  |  |
| Harm | 7.9 | (2.3) | 8.1 | (2.5) | 7.6 | (2.4) | 8.6 | (2.2) | 7.6 | (2.1) |
| Overuse | 11.3 | (3.0) | 11.4 | (3.1) | 10.8 | (3.0) | 12.1 | (2.8) | 10.8 | (3.0) |
| Health anxiety, mean (SD) | 10.8 | (4.9) | 9.7 | (5.0) | 10.1 | (4.7) | 10.4 | (4.5) | 11.5 | (4.9) |

*n=19 missing values (5.8%); ^a^ n=19 missing values (%); ^b^ n=28 missing values (8.6%); ^c^ n=21 missing values (%); ^d^ Menopause hormone therapy; ^e^ n=11 missing values (3.4%); ^f^ n=5 missing values (1.5%)
