# Supplementary material for: Women’s health behaviour change after receiving breast cancer risk estimates with tailored screening and prevention recommendations
Source: BMC Cancer. 2022 Jan 16;22:69. doi: 10.1186/s12885-022-09174-3 (PMC8761310; doi:10.1186/s12885-022-09174-3)
Supplement: Supplementary file 3 — Additional file 3. [file 12885_2022_9174_MOESM3_ESM.docx]

**Supplement 3.** Correspondence of participants’ self-reported and counselled breast cancer risk

|  |  | **Counselled risk**^a^ | | | | | | | | |  |
| --- | --- | --- | --- | --- | --- | --- | --- | --- | --- | --- | --- |
|  |  | Low | | Average | | Moderate | | High | | **Total** |  |
| **Self-reported risk**  N (%^b^) | Low | 30 | (62.5) | 20 | (28.2) | 5 | (6.3) | 2 | (1.9) | 57 | (18.6) |
|  | Average | 17 | (35.4) | 49 | (69.0) | 29 | (36.3) | 23 | (21.5) | 118 | (38.6) |
|  | Moderate | 1 | (2.1) | 2 | (2.8) | 42 | (52.5) | 31 | (29.0) | 76 | (24.8) |
|  | High | 0 | (-) | 0 | (-) | 4 | (5.0) | 51 | (47.7) | 55 | (18.0) |
|  | **Total** | 48 | (100) | 71 | (100) | 80 | (100) | 107 | (100) | 306 | (100) |

^a^ n=19 missing counselled breast cancer risk due to incorrect/missing PROCAS study identification number and/or date of birth; ^b^ Percentages based on actual risk
