# Supplementary material for: Women’s health behaviour change after receiving breast cancer risk estimates with tailored screening and prevention recommendations
Source: BMC Cancer. 2022 Jan 16;22:69. doi: 10.1186/s12885-022-09174-3 (PMC8761310; doi:10.1186/s12885-022-09174-3)
Supplement: Supplementary file 4 — Additional file 4. [file 12885_2022_9174_MOESM4_ESM.docx]

**Supplement 4.** Characteristics of the PROCAS population (n=53,596)

**Supplement 3.** Characteristics of the PROCAS population (n=53,596) *(Continued)*

**From:** Evans, D. G., Astley, S., Stavrinos, P., Harkness, E., Donnelly, L. S., Dawe, S., ... & Howell, A. (2016). PROCAS: Predicting Risk of Breast Cancer at Screening. In Improvement in risk prediction, early detection and prevention of breast cancer in the NHS Breast Screening Programme and family history clinics: a dual cohort study. NIHR Journals Library (Table 32; page 75).
