# Supplementary material for: Women’s health behaviour change after receiving breast cancer risk estimates with tailored screening and prevention recommendations
Source: BMC Cancer. 2022 Jan 16;22:69. doi: 10.1186/s12885-022-09174-3 (PMC8761310; doi:10.1186/s12885-022-09174-3)
Supplement: Supplementary file 5 — Additional file 5. [file 12885_2022_9174_MOESM5_ESM.docx]

**Supplement 5.** Results from the main analysis: univariable and multivariable logistic regression modelling.

**Table 3**. Explorative analyses of factors associated with early detection behaviours after risk feedback with tailored screening recommendations

| **Characteristic** | **Supplemental mammography intent** | | **Increased breast self-examination** | |
| --- | --- | --- | --- | --- |
|  | **Unadjusted** | **Multi-adjusted^a^** | **Unadjusted** | **Multi-adjusted^a^** |
|  | OR (95% CI) | OR (95% CI) | OR (95% CI) | OR (95% CI) |
| Self-reported  breast cancer risk |  |  |  |  |
| Low | n/a | n/a | 0.62 (0.29, 1.30) | 0.66 (0.30, 1.45) |
| Average | Reference | Reference | Reference | Reference |
| Moderate | **4.31** (2.16, 8.60) | **3.88** (1.86, 8.07) | **2.35** (1.31, 4.22) | **2.43** (1.30, 4.53) |
| High | n/a | n/a | **3.98** (2.08, 7.61) | **3.83** (1.89, 7.77) |
| Age (year)^c^ | 1.04 (0.98, 1.10) | 1.03 (0.97, 1.10) | 0.98 (0.93, 1.02) | 0.98 (0.93, 1.03) |
| Education |  |  |  |  |
| Lower | Reference | Reference | Reference | Reference |
| Higher secondary | 0.92 (0.43, 1.96) | 1.03 (0.44, 2.39) | 0.92 (0.49, 1.72) | 0.90 (0.46, 1.74) |
| Higher vocational | 0.69 (0.33, 1.43) | 0.73 (0.32, 1.63) | 0.59 (0.33, 1.07) | 0.54 (0.28, 1.03) |
| FDR^d^ with breast cancer |  |  |  |  |
| No | Reference | Reference | Reference | Reference |
| Yes | **2.01** (1.14, 3.53) | **2.03** (1.02, 4.03) | **1.94** (1.23, 3.04) | 1.10 (0.64, 1.90) |
| Benign breast disease |  |  |  |  |
| No | Reference | Reference | Reference | Reference |
| Yes | 1.37 (0.77, 2.43) | 1.20 (0.65, 2.23) | **1.67** (1.05, 2.64) | 1.46 (0.89, 2.41) |
| Previous breast biopsy |  |  |  |  |
| No | Reference | Reference | Reference | Reference |
| Yes | 1.44 (0.76, 2.70) | 1.56 (0.63, 3.83) | **1.78** (1.07, 2.96) | 1.45 (0.71, 2.95) |
| General health^e^ | 0.99 (0.98, 1.01) | 0.99 (0.97, 1.01) | 0.99 (0.97, 1.00) | 0.99 (0.97, 1.01) |
| Health anxiety^f^ | 0.99 (0.93, 1.05) | 0.99 (0.92, 1.07) | 1.04 (1.00, 1.09) | 1.01 (0.95, 1.07) |
| Health locus of control^g^ |  |  |  |  |
| No preference | Reference | Reference | Reference | Reference |
| Internal | 1.13 (0.40, 3.15) | 0.94 (0.31, 2.83) | 1.07 (.047, 2.40) | 1.18 (0.49, 2.86) |
| Chance | 1.50 (0.57, 3.95) | 1.12 (0.39, 3.20) | 1.82 (0.85, 3.90) | 1.99 (0.87, 4.57) |

^a^ Adjusted for age, education, first degree relative with breast cancer, benign breast disease, general health, breast cancer risk, and health anxiety; ^b^ Odds ratios in bold are significant with p<0.05; ^c^ Age per 1 year increase; ^d^ FDR = first degree relative; ^e^ General health per one point increase; ^f^ Health anxiety per one point increase; ^g^ n=11 people with a physician health locus of control were excluded due to small sample size

**Table 4**. Explorative analyses of factors associated with preventive behaviours after breast cancer risk feedback with tailored prevention recommendations

| **Characteristic** | **Started medication** | | **Changed diet** | | **Increased exercise** | | **Limited alcohol intake** | |
| --- | --- | --- | --- | --- | --- | --- | --- | --- |
|  | **Unadjusted** | **Multi-adjusted**^a^ | **Unadjusted** | **Multi-adjusted**^a^ | **Unadjusted** | **Multi-adjusted**^a^ | **Unadjusted** | **Multi-adjusted**^a^ |
|  | OR (95% CI) | OR (95% CI) | OR (95% CI) | OR (95% CI) | OR (95% CI) | OR (95% CI) | OR (95% CI) | OR (95% CI) |
| Self-reported  breast cancer risk |  |  |  |  |  |  |  |  |
| Low | n/a | n/a | 0.74^h^ (0.29, 1.86) | 0.79^h^ (0.29, 2.16) | 1.44 (0.71, 2.90) | 1.71 (0.80, 3.66) | 1.17^h^ (0.54, 2.56) | 1.22^h^ (0.54, 2.77) |
| Average | n/a | n/a | Reference | Reference | Reference | Reference | Reference | Reference |
| Moderate | **0.24**^h^ (0.10, 0.58) | **0.12**^h^ (0.04, 0.39) | **2.39**^b^ (1.21, 4.74) | **2.57** (1.19, 5.55) | 0.98 (0.49, 1.94) | 0.89 (0.43, 1.85) | 1.26 (0.62, 2.56) | 1.26 (0.60, 2.64) |
| High | Reference | Reference | **4.57** (2.26, 9.24) | **4.60** (2.03, 10.42) | **2.59** (1.33, 5.05) | **2.18** (1.05, 4.56) | 1.43 (0.67, 3.03) | 1.44 (0.64, 3.24) |
| Age^i^ | 1.09 (0.99, 1.19) | **1.13** (1.01, 1.28) | 0.98 (0.93, 1.03) | 0.98 (0.92, 1.05) | 1.01 (0.95, 1.06) | 1.01 (0.96, 1.07) | 1.01 (0.95, 1.07) | 1.01 (0.95, 1.07) |
| Education |  |  |  |  |  |  |  |  |
| Lower | 0.77^h^ (0.25, 2.31) | 0.61^h^ (0.15, 2.41) | Reference | Reference | Reference | Reference | Reference | Reference |
| Higher secondary | 1.88 (0.70, 5.00) | 2.05 (0.64, 6.62) | 1.09 (0.54, 2.23) | 1.42 (0.63, 3.22) | 0.91 (0.46, 1.80) | 1.08 (0.52, 2.23) | 1.14 (0.53, 2.42) | 1.14 (0.53, 2.49) |
| Higher vocational | Reference | Reference | 0.90 (0.45, 1.82) | 1.13 (0.50, 2.55) | 0.93 (0.48, 1.79) | 1.11 (0.55, 2.27) | 0.79 (0.38, 1.65) | 0.78 (0.36, 1.67) |
| BMI^j^ | 0.98 (0.87, 1.11) | 0.98 (0.84, 1.14) | **1.22** (1.12, 1.32) | **1.24** (1.13, 1.36) | **1.11** (1.03, 1.20) | **1.10** (1.01, 1.19) | 0.99 (0.92, 1.08) | 0.99 (0.91, 1.08) |
| FDR^c^ breast cancer |  |  |  |  |  |  |  |  |
| No | Reference | Reference | Reference | Reference | Reference | Reference | Reference | Reference |
| Yes | 0.84 (0.34, 2.05) | 0.42 (0.13, 1.37) | **2.04** (1.21, 3.42) | 1.11 (0.57, 2.13) | **1.72** (1.05, 2.83) | 1.70 (0.93, 3.11) | 1.12 (0.65, 1.93) | 1.05 (0.56, 1.98) |
| Benign breast disease |  |  |  |  |  |  |  |  |
| No | Reference | Reference | Reference | Reference | Reference | Reference | Reference | Reference |
| Yes | 0.60 (0.25, 1.40) | 0.83 (0.29, 2.37) | 1.09 (0.64, 1.86) | 0.88 (0.47, 1.64) | 0.80 (0.48, 1.35) | 0.75 (0.43, 1.30) | 1.05 (0.59, 1.85) | 1.02 (0.57, 1.83) |
| Previous breast biopsy |  |  |  |  |  |  |  |  |
| No | Reference | Reference | Reference | Reference | Reference | Reference | Reference | Reference |
| Yes | 1.49 (0.59, 3.77) | 1.76 (0.31, 9.89) | 1.12 (0.63, 2.00) | 1.31 (0.55, 3.16) | 0.79 (0.44, 1.43) | 0.90 (0.40, 2.00) | 1.53 (0.83, 2.82) | 2.01 (0.83, 4.91) |
| Co-morbidity |  |  |  |  |  |  |  |  |
| 0-1 | Reference | Reference | Reference | Reference | Reference | Reference | Reference | Reference |
| ≥ 2 | 0.74 (0.32, 1.68) | 1.37 (0.48, 3.93) | 1.64 (0.97, 2.75) | 1.42 (0.75, 2.70) | 0.76 (0.46, 1.24) | 0.62 (0.35, 1.11) | 0.81 (0.47, 1.39) | 0.77 (0.42, 1.41) |
| General health^k^ | 1.02 (0.99, 1.05) | 1.00 (0.95, 1.04) | **0.98** (0.97, 1.00) | 1.00 (0.98, 1.02) | 0.98 (0.97, 1.00) | 0.99 (0.97, 1.01) | 1.00 (0.98, 1.02) | 1.00 (0.98, 1.03) |
| Life events |  |  |  |  |  |  |  |  |
| 0-1 | Reference | Reference | Reference | Reference | Reference | Reference | Reference | Reference |
| ≥ 2 | 0.50 (0.20, 1.28) | 0.47 (0.15, 1.41) | 0.69 (0.38, 1.27) | 0.63 (0.32, 1.22) | 0.96 (0.55, 1.67) | 0.92 (0.52, 1.64) | 0.54 (0.27, 1.06) | 0.53 (0.27, 1.06) |

**Table 4**. *Continued*

| **Characteristic** | **Started medication** | | **Changed diet** | | **Increased exercise** | | **Limited alcohol intake** | |
| --- | --- | --- | --- | --- | --- | --- | --- | --- |
|  | **Unadjusted** | **Multi-adjusted**^c^ | **Unadjusted** | **Multi-adjusted**^c^ | **Unadjusted** | **Multi-adjusted**^c^ | **Unadjusted** | **Multi-adjusted**^c^ |
|  | OR (95% CI) | OR (95% CI) | OR (95% CI) | OR (95% CI) | OR (95% CI) | OR (95% CI) | OR (95% CI) | OR (95% CI) |
| Health anxiety^L^ | 0.91 (0.84, 1.00) | 0.88 (0.77, 1.00) | **1.06** (1.01, 1.12) | 1.04 (0.97, 1.12) | 1.02 (0.97, 1.07) | 1.01 (0.94, 1.07) | 1.00 (0.95, 1.06) | 1.00 (0.94, 1.08) |
| Current medication use^f^ |  |  |  |  |  |  |  |  |
| No | Reference | Reference | n/a | n/a | n/a | n/a | n/a | n/a |
| Yes | 1.41 (0.60, 3.31) | 1.09 (0.38, 3.18) | n/a | n/a | n/a | n/a | n/a | n/a |
| Beliefs about medicines^g^ |  |  |  |  |  |  |  |  |
| Harm | 0.87 (0.71, 1.06) | 0.92 (0.72, 1.17) | n/a | n/a | n/a | n/a | n/a | n/a |
| Overuse | 0.88 (0.76, 1.02) | 0.89 (0.74, 1.06) | n/a | n/a | n/a | n/a | n/a | n/a |

^a^ Adjusted for age, education, BMI, first degree relative with breast cancer, benign breast disease, general health, breast cancer risk, and health anxiety; ^b^ Odds ratios in bold are significant with p<0.05; ^c^ FDR = first degree relative; ^d^ n=11 women with a physician locus of control were excluded due to small sample size; ^e^ Not available due to small subgroups (n<10); ^f^ Additionally adjusted for beliefs about medicines; ^g^ Additionally adjusted for current medication use; ^h^ Estimate based on a subgroup with fewer than 10 study participants; ^i^ Age per 1 year increase; ^j^ BMI per 1 point increase; ^k^ General health per one point increase; ^L^ Health anxiety per one point increase
